# Diversification of aminoacyl-tRNA synthetase activities *via* genomic duplication

**DOI:** 10.3389/fphys.2022.983245

**Published:** 2022-08-19

**Authors:** Natalie Krahn, Dieter Söll, Oscar Vargas-Rodriguez

**Affiliations:** ^1^ Department of Molecular Biophysics and Biochemistry, Yale University, New Haven, CT, United States; ^2^ Department of Chemistry, Yale University, New Haven, CT, United States

**Keywords:** gene duplication, aminoacyl-tRNA synthetase, evolution, translation, tRNA, noncanonical functions, genetic code

## Abstract

Intricate evolutionary events enabled the emergence of the full set of aminoacyl-tRNA synthetase (aaRS) families that define the genetic code. The diversification of aaRSs has continued in organisms from all domains of life, yielding aaRSs with unique characteristics as well as aaRS-like proteins with innovative functions outside translation. Recent bioinformatic analyses have revealed the extensive occurrence and phylogenetic diversity of aaRS gene duplication involving every synthetase family. However, only a fraction of these duplicated genes has been characterized, leaving many with biological functions yet to be discovered. Here we discuss how genomic duplication is associated with the occurrence of novel aaRSs and aaRS-like proteins that provide adaptive advantages to their hosts. We illustrate the variety of activities that have evolved from the primordial aaRS catalytic sites. This precedent underscores the need to investigate currently unexplored aaRS genomic duplications as they may hold a key to the discovery of exciting biological processes, new drug targets, important bioactive molecules, and tools for synthetic biology applications.

## Introduction

Aminoacyl-tRNA synthetases (aaRSs) catalyze one of the most consequential reactions during mRNA translation: the ligation of amino acids to their cognate tRNAs. Except for selenocysteine, there is a dedicated aaRS family for each proteinogenic amino acid. These families are sorted into two almost equally populated classes (class I and II) based on the architecture of their catalytic site, their mechanism of tRNA aminoacylation, and their phylogenetic relationship (Cusack et al., 1990; Eriani et al., 1990; Ribas de Pouplana and Schimmel, 2001b; Zhang et al., 2006). Synthetases catalyze tRNA aminoacylation in a two-step reaction wherein the amino acid is first condensed with ATP, to form an aminoacyl-adenylate intermediate, and subsequently esterified to the 3′-end adenosine of the tRNA. The efficiency and specificity of aaRSs are paramount for the accurate and productive translation of genomic information into proteins.

aaRSs are multi-domain enzymes consisting of a conserved ancient catalytic domain and additional accessory domains that increase their specificity and/or efficiency. (Guo et al., 2010; Zhang et al., 2021). Common features of aaRSs include tRNA binding domains and hydrolytic (or editing) domains that facilitate tRNA recognition and correct aminoacylation errors, respectively (Ling et al., 2009). aaRSs have also expanded their biological function beyond tRNA aminoacylation by adding new domains or motifs (Wolf et al., 1999; Schimmel and Ribas De Pouplana, 2000; Guo and Schimmel, 2013; Pang et al., 2014; Kwon et al., 2019). This is particularly prevalent in aaRSs from higher organisms (Guo et al., 2010). aaRSs originated early, and consequently, have a complex evolutionary history that contributed to the structural and biochemical diversification of each aaRS family (Wolf et al., 1999; Ribas de Pouplana and Schimmel, 2000; Woese et al., 2000; Ribas de Pouplana and Schimmel, 2001a; Al-Shayeb et al., 2020).

**FIGURE 1:**
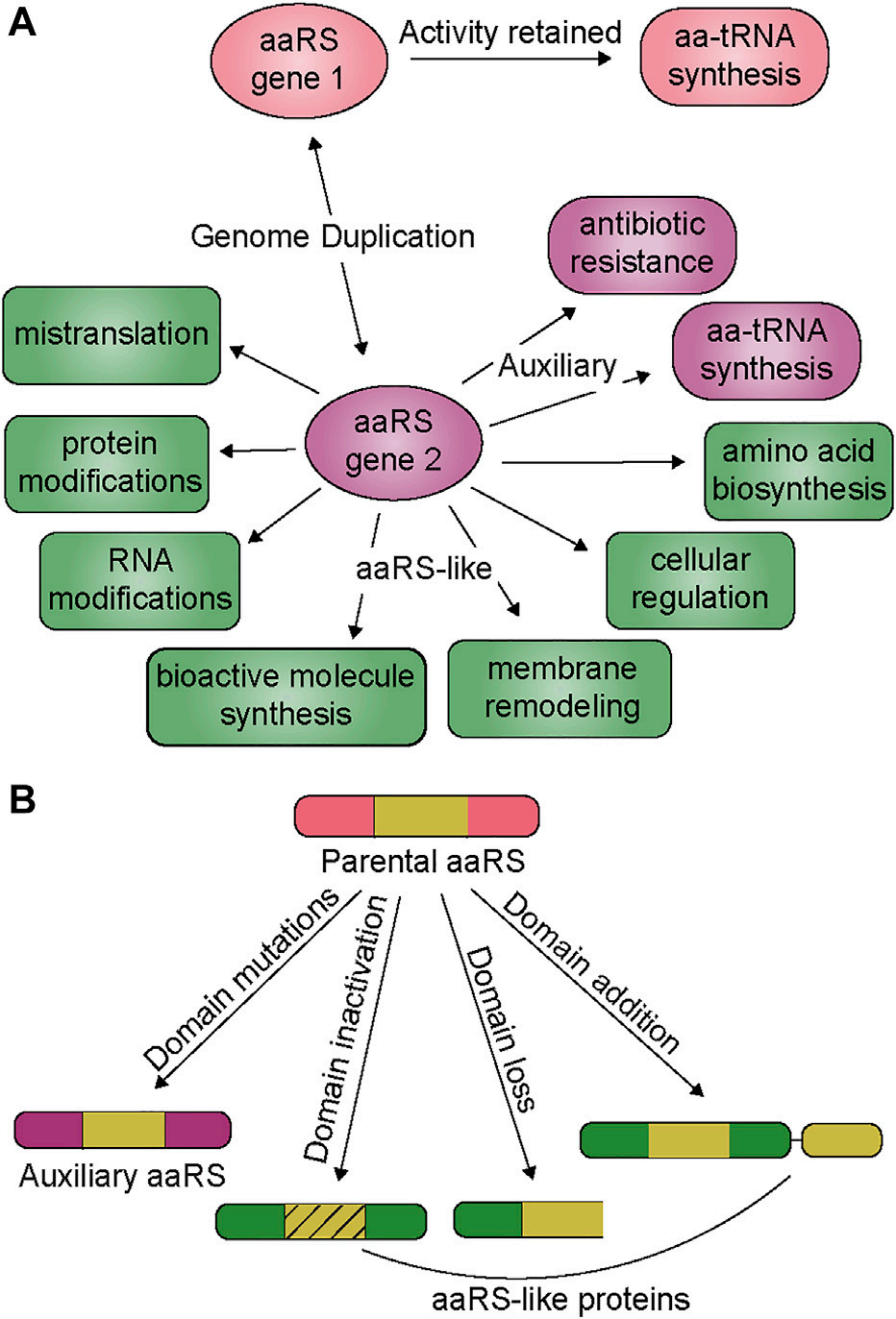
**(A)** Duplication and divergence of aaRS genes. Genomic duplication generates a new aaRS gene (aaRS gene 2) while preserving the parental copy (aaRS gene 1) which is responsible for the housekeeping tRNA aminoacylation activity. The second copy (aaRS gene 2) either develops new characteristics under specific selection pressures (auxiliary function, purple rounded squares) or a combination of genetic drift and selection can produce an aaRS-like protein with new activity (green boxes) **(B)** From the parental aaRS protein, mutations and protein architecture can change, leading to non-canonical functions. Domain mutations generally give rise to auxiliary functions while aaRS-like proteins are found with inactive domains, or the loss or addition of domains.

In many organisms, the number of aaRS genes can be higher than that of the genetically encoded amino acids, which is the consequence of apparent genomic duplication of aaRSs for a particular amino acid (Rubio et al., 2015; Chaliotis et al., 2017). The duplicated aaRSs generally share a conserved tertiary structure but with low sequence homology, and distinct evolutionary origins. Thus, acquisition of additional genes is likely possible *via* horizontal gene transfer (HGT) or duplicated within a single domain. These evolutionary events can occur separately or simultaneously to accelerate the emergence of aaRSs with new or improved functions (Conant and Wolfe, 2008; Innan and Kondrashov, 2010; Treangen and Rocha, 2011). The evolutionary drive for genomic duplication of aaRSs is an organism’s response to physical forces and natural selection, influenced by their environment and lifestyle. In this review we describe the functional outcome of genomic aaRS duplications and highlight the broad range of additional functions imparted by these evolved aaRSs, from maintaining aminoacylation activity under stress to regulation of cell cycle, antibiotic resistance, RNA and protein modifications, and mistranslation (Figure 1 and Table 1). We discuss how these events are not rare, fortuitous occurrences, but rather are found repeatedly throughout evolution. Given the large number of organisms with additional aaRS genes, we surmise that many new and exciting functions can be uncovered by investigating this phenomenon. Our focus is on genes which retained their catalytic domain and have a clearer connection to their evolution from a gene duplication event. Other reviews provide more details on genes which are related to the tRNA binding domain or editing domain (Francklyn, 2005; Giegé and Springer, 2016).

**TABLE 1:** List of duplicated aminoacyl-tRNA synthetases and their evolved function.

aaRS	Auxiliary function	Paralog function
AlaRS	—	(a) aa:CP ligases add Ala to Ppant which is linked to a carrier protein (Mocibob et al., 2013)
AspRS	—	(a) AS-A/AS-AR synthesizes l-Asp (Nakamura et al., 1981; Nakatsu et al., 1998)
		(b) ErdS catalyzes synthesis of Erg-Asp (Fields and Roy, 2018; Yakobov et al., 2020)
CysRS	—	(a) CysRS* inserts Cys at opal (UGA) codons (Mukai et al., 2017b)
		(b) MhC catalyzes Cys ligation onto GlN-Ins in MHS biosynthesis (Sareen et al., 2002; Tremblay et al., 2008)
GluRS	—	(a) YadB (Glu-Q-RS) transfers Glu onto queuosine of anticodon in Asp-tRNA^Asp^ (Blaise et al., 2004; Dubois et al., 2004; Salazar et al., 2004)
GlyRS	(a) GlyRS2 produces Gly-tRNA^Gly^ at high temperatures (Chen et al., 2012)	(a) aa:CP ligases add Gly to Ppant which is linked to a carrier protein (Mocibob et al., 2013)
HisRS	—	(a) HisZ synthesizes l-His (Sissler et al., 1999; Thomson et al., 2019)
IleRS	(a) IleRS2 is resistant to mupirocin (Zanki et al., 2022)	(a) SbzA transfers Ile onto altemicidin (Hu et al., 2019)
LeuRS	(a) LeuRS-I produces leucyl-adenylates (Weitzel et al., 2020)	—
	(b) LeuRS2 produces low levels of Leu-tRNA^Leu^ (Fang et al., 2014)	
LysRS	—	(a) LysU produces Lys-tRNA^Lys^ under stress (Brevet et al., 1995)
		(b) PoxA (GenX, YjeA) transfers β-lysine onto EF-P (Yanagisawa et al., 2010; Roy et al., 2011)
		(c) LysX transfers Lys to peptidoglycan (Maloney et al., 2009)
ProRS	—	(a) ProRSx inserts Pro at Ala codons (Vargas-Rodriguez et al., 2021)
PylRS	—	(a) PylRS2 aminoacylates cognate tRNA^Pyl^ (Zhang et al., 2022)
SerRS	(a) SerRS2 is resistant to albomycin (Zhou et al., 2019)	(a) SLIMP regulates cell-cycle progression (Picchioni et al., 2019)
		(b) aa:CP ligases add Ser to Ppant which is linked to a carrier protein (Mocibob et al., 2010)
ThrRS	(a) T2 produces Ser-tRNA^Thr^ in low zinc conditions (Rubio et al., 2015)	(a) ThrRS-L plays a role in the MSC and recycles tRNA^Thr^ for ThrRS under stress (Zhou et al., 2019)
	(b) ThrRS-L produces Thr-tRNA^Thr^ but with poor editing activity (Zhou et al., 2013)	
TrpRS	(a) TrpRSII nitrates Trp on Trp-tRNA^Trp^ in toxic environments (Buddha et al., 2004a; Buddha et al., 2004b)	—
	(b) TrpRS1 is resistant to indolmycin (Kitabatake et al., 2002; Vecchione and Sello, 2009)	
TyrRS	(a) TyrZ produces Tyr-tRNA^Tyr^ with high selectivity for l-Tyr under stress (Williams-Wagner et al., 2015)	—
	(b) Two fused TyrRSs produce 1 or 2 Tyr-tRNA^Tyr^ (Duchêne et al., 2005; Larson et al., 2011)	

## Auxiliary tRNA aminoacylation

### tRNA aminoacylation under pressure

The capacity to acclimate to environmental changes is vital for most organisms, particularly in conditions that jeopardize cellular homeostasis and cause cell death. Cells generally respond to environmental cues by expressing dedicated factors to counteract a given stress. In several species, genomic duplication of aaRSs offers a mechanism to endure challenges such as disturbances in amino acid concentration, metal salts, temperature, and exposure to toxic substances. For example, *Bacillus subtilis* encodes a specialized tyrosyl-tRNA synthetase (TyrZ) to protect cells against high concentrations of d-Tyr and possibly other nonproteinogenic amino acids (Williams-Wagner et al., 2015). TyrZ accomplishes this through its increased selectivity for l-Tyr over d-Tyr (compared to the housekeeping TyrS) preventing misincorporation of d-Tyr into proteins. The physiological conditions that control TyrZ expression remain unknown.

In the green-blue alga *Anabaeana* sp. PCC7120, low zinc levels cause dissociation and inactivation of the constitutively expressed threonyl-tRNA synthetase (ThrRS-T1). This restrictive condition induces expression of a second ThrRS gene, T2. In contrast to T1, T2 can dimerize in low zinc concentrations and maintain its aminoacylation activity (Rubio et al., 2015). This could provide an alternate strategy for organism viability under low zinc conditions.

Gram-positive bacteria have adopted a similar approach to acclimate to their environment through a copy of tryptophanyl-tRNA synthetase (TrpRS II) that is induced upon radiation damage. One role of TrpRS II is its ability to reduce nitric oxide toxicity by interacting with nitric oxide synthase (NOS) (Buddha et al., 2004a). While retaining Trp aminoacylation activity, TrpRS II harnesses NOS to catalyze regioselective nitration of Trp (Buddha et al., 2004b). It remains unclear whether nitro-tryptophan is used by the ribosome for protein synthesis or whether it plays a role in DNA repair.

*Saccharomyces cerevisiae* and *Vanderwaltozyma polyspora* have also adapted to environmental strains with an additional copy of glycyl-tRNA synthetase (GlyRS2). Under standard conditions, GlyRS2 has ∼5-fold lower activity relative to GlyRS1 (the housekeeping enzyme) but is able to rescue the impaired activity of GlyRS1 under stress (e.g. high temperature) (Chen et al., 2012). It is hypothesized that *Candidatus* Methanohalarchaeum thermophilum HMET1 has evolved an additional pyrrolysyl-tRNA synthetase (PylRS2) for a similar purpose. Unlike GlyRS2, PylRS2 has its own cognate tRNA^Pyl^2 and is shown to be orthogonal to PylRS1/tRNA^Pyl^1. Therefore, it is also possible that both PylRS systems are expressed simultaneously (Zhang et al., 2022).

### Antibiotic resistance

The potent antibiotics albomycin, mupirocin, and indolmycin inhibit protein synthesis by targeting the activities of seryl-tRNA synthetase (SerRS), isoleucyl-tRNA synthetase (IleRS), and TrpRS, respectively (Montgomery et al., 2015). These antibiotics are produced by bacteria that avoid suicide by encoding a second gene copy of the corresponding aaRS (SerRS, IleRS, or TrpRS) that is insensitive to the action of the related antibiotic. The coexisting aaRS genes are evolutionarily distinct from each other, exhibiting low sequence homology (∼30% sequence identity) and different biochemical characteristics (Zeng et al., 2009). They also display devoted expression patterns where the antibiotic-resistant aaRS is expressed primarily when synthesis of the antibiotic is active while the other acts as the housekeeping enzyme (Kitabatake et al., 2002; Vecchione and Sello, 2009).

In addition to facilitating the synthesis of antibiotics, acquisition of supplementary aaRS genes to gain antibiotic resistance has been observed in strains of the relevant bacterial human pathogens *Staphylococcus aureus* and *Bacillus anthracis*. These strains have acquired a plasmid encoded IleRS that is insensitive to mupirocin (Hodgson et al., 1994). Barring the low activity of IleRS2, its retained editing capacity and amino acid specificity compensates for the sensitivity of IleRS1 to mupirocin (Brown et al., 2003; Zanki et al., 2022).

### Diverging tRNA aminoacylation functions

In many cases, the role of duplicated aaRS genes is not yet well understood. For instance, the additional leucyl-tRNA synthetase (LeuRS-I) in species from the archaeal family *Sulfolobaceae* is critical for optimal cell growth (Weitzel et al., 2020). LeuRS-I contains a disrupted CPI editing domain and a very divergent acidic C-terminal domain. Surprisingly, although LeuRS-I can bind tRNA^Leu^ and produce a leucyl-adenylate, it is unable to aminoacylate. LeuRS duplication in Halobacteria (LeuRS2), evolved an enzyme with drastically reduced aminoacylation activity but preserved the affinity for tRNA^Leu^ (Fang et al., 2014). The functional and regulatory mechanisms of LeuRS2 also remain unknown. The remarkable characteristics of these LeuRS genes suggest they play a role outside of protein synthesis, possibly mediating cellular functions in a tRNA-dependent manner.

A genomic aaRS duplication found in trypanosomes encodes a tyrosyl-tRNA synthetase (TyrRS) gene consisting of two independent TyrRSs. In each TyrRS enzyme, one of the domains has lost activity, giving rise to a pseudo-dimer. This pseudo-dimer is capable of only one aminoacylation reaction, though it is twice as large as a single TyrRS enzyme (Larson et al., 2011). The function of this pseudo-dimer remains unclear. A similar occurrence is found in *Arabidopsis thaliana*, but in this case both TyrRS proteins appear to be fully active synthetases, each containing both a ‘HIGH’ and ‘KMSK’ motif (Duchêne et al., 2005). The duplication in these organisms is suggested to have occurred later in evolution as additional mutagenesis has not yet inactivated a domain (Larson et al., 2011).

As these additional aaRSs continue to evolve, their functions begin to deviate from canonical aminoacylation towards synthetase-like proteins. Threonyl-tRNA synthetase-like protein (ThrRS-L) is an example found in higher eukaryotes, that retains aminoacylation activity, but its low expression levels and poor editing activity suggests it did not evolve for protein translation. Instead its N-terminal extension (Zheng et al., 2006) targets ThrRS-L to the multi-synthetase complex (Zhou et al., 2013) where it is hypothesized to play a role in the recycling of tRNA^Thr^ for ThrRS under stress conditions (Zhou et al., 2019).

## Aminoacyl-tRNA synthetase-like proteins

### Amino acid biosynthesis

The active sites of aaRSs offer amenable scaffolds that can be co-opted for alternative functions involving ATP-dependent and/or amino acid-related reactions. Consequently, aaRS-like proteins have evolved to participate in amino acid biosynthesis. In some bacteria and archaea, an aspartyl-tRNA synthetase (AspRS)-like enzyme, asparagine synthetase A (AS-A), is responsible for l-asparagine biosynthesis (Nakamura et al., 1981; Roy et al., 2003). Like AspRS, AS-A activates aspartate using ATP, however, the amino acid is transferred to an acceptor ammonia instead of a tRNA due to the absent tRNA binding domain (Nakamura et al., 1981; Nakatsu et al., 1998). AS-A presumably descends from gene duplication of an ancestral archaeal AspRS that also gave rise to extant canonical asparginyl-tRNA synthetase and was eventually transferred to bacteria *via* HGT (Roy et al., 2003). Notably, two additional pathways for asparagine biosynthesis exist. Another direct pathway catalyzed by the glutamine-dependent asparagine synthetase B and an indirect pathway involving the conversion of the Asp-tRNA^Asn^ to Asn-tRNA^Asn^ by GatCAB transamidase (Francklyn, 2003; Sheppard et al., 2008). The latter mechanism may constitute the original route to asparagine as it relies on an additional, non-discriminating AspRS attaching Asp to tRNA^Asn^ (Becker and Kern, 1998; Min et al., 2002; Francklyn, 2003).

HisZ, a histidyl-tRNA synthetase paralog, is also involved in amino acid biosynthesis. HisZ acts as a functional regulatory subunit of the ATP-phosphoribosyl-transferase (HisG), which catalyzes the first step of histidine biosynthesis (Sissler et al., 1999). In contrast to AS-A, HisZ is only found in bacteria and does not possess adenylation activity; instead, it mediates the allosteric inhibition of His biosynthesis in the presence of His (Vega et al., 2005; Thomson et al., 2019).

### Cell-cycle regulation and signaling

In insects, a conserved SerRS paralog, known as SLIMP (SerRS-like insect mitochondrial protein), has evolved as a key regulator of mitochondrial protein synthesis and DNA replication. SLIMP prevents mitochondrial DNA accumulation by association with LON protease while also forming a heterodimer with canonical mitochondrial SerRS (Picchioni et al., 2019), an essential function for cell-cycle progression. SLIMP possibly originated via duplication of mitochondrial SerRS, retaining tRNA binding capabilities specific for mitochondrial tRNA^Ser^ but lacking aminoacylation activity (Guitart et al., 2010).

In *Escherichia coli*, LysU, an additional lysyl-tRNA synthetase (LysRS), is induced under stress conditions including anaerobiosis, heat shock, oxidative stress, or low external pH (Hirshfield et al., 1981; Lévêque et al., 1991; Ito et al., 1993). While LysU is capable of tRNA aminoacylation (Brevet et al., 1995), it is found to have multiple roles outside translation. For example, LysU functions in the synthesis of alarmone diadenosine 5′,5‴-P1,P4-tetraphosphate (Ap_4_A) (Blanquet et al., 1983; Wright et al., 2006; Chen et al., 2013) and capping of the 5′-end of RNA transcripts by Ap_4_ (Luciano et al., 2019). Accumulation of Ap_4_A ultimately leads to cell death while Ap_4_-capped RNAs have prolonged half-lives. Therefore, LysU is indirectly involved in both cellular regulation and gene expression, respectively (Ji et al., 2019; Luciano et al., 2019).

### Post-transcriptional modification

Synthetase paralogs have also evolved as RNA modifiers. Glutamyl-queuosine tRNA^Asp^ synthetase (Glu-Q-RS) activates Glu in the absence of tRNA and attaches it onto the queuosine in the first position of the anticodon of tRNA^Asp^ (Blaise et al., 2004; Dubois et al., 2004; Salazar et al., 2004). Glu-Q-RS, also known as YadB, is present in proteobacteria, cyanobacteria, and actinobacteria and is homologous to the catalytic domain of glutamyl-tRNA synthetase, while lacking an anticodon binding domain (Salazar et al., 2004). The role and essentiality of Glu-Q-RS in these organisms remains unclear, however it does provide more information regarding the evolutionary pathway of the non-essential Glu-Q-RS and its conservation across different bacterial genera (Ravishankar et al., 2016).

### Post-translational modification

Other aaRS mimics have been found to modify proteins. PoxA (also known as GenX and YjeA) is a paralog of LysRS that modifies elongation factor-P (EF-P) post-translationally with an amino acid (Yanagisawa et al., 2010). Although PoxA is capable of acylating both α-lysine and β-lysine onto EF-P, it prefers the latter, thereby creating an orthogonal system to the natural LysRS (Roy et al., 2011). This modification on EF-P, analogous to modification of the eukaryotic homolog eIF5A with hypusine, is suggested to play a role for *Salmonella* to establish virulence and maintain a stress resistance phenotype (Navarre et al., 2010).

Another family of aaRS-related post-translational modification enzymes is the amino acid:carrier protein (aa:CP) ligase. These ligases from methanogenic archaea attach an amino acid onto 4′-phosphopantetheine (Ppant) which is linked to a CP. aa:CP ligases are homologs of class II aaRSs which have lost their tRNA-binding domain and canonical tRNA aminoacylation activity (Mocibob et al., 2010). They still act as dimers and are dependent on zinc for their catalytic activity, however their mode of macromolecular recognition is distinct from aaRSs. Instead, their catalytic strategy is reminiscent of adenylation domains: activation of the amino acid followed by transfer to the Ppant chain. The biological role of amino acid attachment to CPs remains unknown (Mocibob et al., 2013).

### Alternative expression of the genetic code

Recent studies have uncovered novel noncanonical aaRSs that have co-evolved with unique tRNA partners. These aaRS homologs maintained the amino acid specificity of their predecessors while developing affinity for new tRNA substrates. For instance, ProRSx appeared from a genomic duplication of bacterial prolyl-tRNA synthetase in a group of *Streptomyces* species that includes pathogens that cause the common scab disease in staple food crops, particularly in potatoes. ProRSx co-evolved with a unique proline tRNA (tRNA^ProA^) with Ala anticodon. This synthetase ligates Pro to tRNA^ProA^, leading to mistranslation of Ala codons with Pro (Vargas-Rodriguez et al., 2021). Thus, organisms encoding these genes have the capacity to produce multiple variants of the same protein from a single gene by deliberately mistranslating their genetic code. However, the biological function of the ProRSx and tRNA^ProA^ pair is still unknown.

Another example is found in a subgroup of *Desulfobacterales* bacteria that encodes CysRS^*^, a noncanonical cysteinyl-tRNA synthetase (CysRS). CysRS* is genetically coupled with homologs of SelC and SelB (SelC* and SelB*, respectively), which coexist with the wildtype SelC and SelB (Mukai et al., 2017b). CysRS^*^ lacks an anticodon binding domain and contains mutations that enable exclusive aminoacylation of SelC*. The aminoacylated SelC* tRNA incorporates Cys at selenocysteine UGA codons. CysRS* and SelC* are posited to serve as an alternative mechanism for the synthesis of selenoproteins under conditions in which selenium is scarce (Mukai et al., 2017b). These examples add to the growing wealth of evidence that demonstrate the flexibility of the genetic code and how mistranslation can be employed as an adaptive mechanism (Pan, 2013; Ribas de Pouplana et al., 2014).

### Bioactive molecule synthesis

aaRS-like proteins are also involved in the synthesis of important metabolic and bioactive molecules including the antioxidant mycothiol (Newton et al., 2008), and antibiotics albonoursin (Fukushima et al., 1973) and SB-203207 (Stefanska et al., 2000). The CysRS-like protein, MhC, catalyzes the ATP-dependent ligation of Cys to 1-*O* -(2-amino-2-deoxy-α-d-glucopyranosyl)-d-*myo-*inosityl (GlcN-Ins) in the penultimate step of the mycothiol biosynthesis (Sareen et al., 2002; Tremblay et al., 2008). Mycothiol is the major thiol found in actinobacteria acting as a glutathione substitute, the dominant thiol in other bacteria and eukaryotes but absent in actinobacteria (Newton et al., 2008). In *Streptomyces* sp. NCIMB 40513, the final step of the SB-203207 biosynthesis is catalyzed by SbzA, an IleRS homolog. SbzA catalyzes the transfer of Ile from Ile-tRNA^Ile^ onto a non-peptide secondary metabolite during the synthesis of altemicidin (Hu et al., 2019). A similar mechanism of amino acid transfer is observed in a family of enzymes known as cyclodipeptide synthases (CDPs) (Gondry et al., 2009; Yao et al., 2018). CDPs are involved in biosynthetic pathways of diketopiperazines (DKPs) through the formation of two successive peptide bonds. One example is *Streptomyces noursei* AlbC which uses Phe-tRNA^Phe^ and Leu-tRNA^Leu^ as substrates to synthesize Albonoursin, an antibacterial DKP. AlbC does not possess a C-terminal tRNA-binding domain, however its N-terminal domain is structurally similar to TyrRS and TrpRS (Sauguet et al., 2011).

### Membrane remodeling

Membrane remodeling is a crucial biological process that allows cells from all domains of life to navigate in different environments. A recent study found a tRNA-dependent lipid modification process in fungi, which is orchestrated by a single enzyme, ergosteryl-3β-*O*-l-aspartate synthase (ErdS) (Yakobov et al., 2020). In bacteria, membrane glycerolipids are aminoacylated in a tRNA-dependent fashion by aminoacyl-tRNA transferases belonging to the *Domain of Unknown Function 2,156* (DUF2156) family (Fields and Roy, 2018). ErdS is unique in that it comprises catalytic activities from both AspRS and DUF2156; catalyzing attachment of Asp to tRNA^Asp^ and the transfer of the amino acid to ergosterol to produce ergosteryl-3β-*O*-l-aspartate (Erg-Asp), respectively. The evolution of ErdS has been suggested to be important in fungal membrane remodeling, trafficking, antimicrobial resistance, or pathogenicity (Yakobov et al., 2020). *Mycobacterium tuberculosis* has also evolved a two-domain aaRS, LysX, for production of lysinylated phosphatidylglycerol (L-PG). LysX is composed of LysRS fused to an MprF domain, functioning in two biochemical steps to transfer Lys to PG. The production of L-PG works to polarize the membrane, acting as an important frontline defense against invading pathogens (Maloney et al., 2009).

## Discussion

The motivation behind this review is to bring attention to the important biological role of duplication, divergence, and lateral transfer in the functional diversification and innovation of aaRS and aaRS-like proteins. Here we summarized the wide range of functions associated with aaRS duplication involving 15 of the 21 canonical aaRS families (Figure 1 and Table 1). Recent bioinformatic surveys estimated that approximately 95% of sequenced genomes have at least one instance of aaRS genomic duplication encompassing all aaRS families (Rubio et al., 2015; Chaliotis et al., 2017). Most of these genes are yet to be characterized and many of the characterized aaRS genes remain poorly understood. We envision that investigation of aaRS genomic duplication may uncover many unexpected new functions that will contribute to our biological understanding of various species. The use of aaRS duplication as a mechanism to resist, persist and adapt to stresses can shed light on pathogen interactions with their host environments. Notably, many additional aaRS gene copies are primarily encoded by bacteria (possibly due to their predisposition to readily acquire genomic material from other species); thus, they may be targeted for the development of antimicrobials. The involvement of aaRSs in antibiotic biosynthesis (Garg et al., 2008) and resistance should also inspire investigation of aaRS duplication for the discovery of new natural antibiotics. Lastly, several synthetic organisms with expanded genetic alphabets or open codons for reassignment are now available (Malyshev et al., 2014; Fredens et al., 2019). However, the discovery and engineering of new orthogonal aaRS-tRNA pairs to expand the genetic code of these organisms for non-canonical amino acid insertion into proteins is imperative (Vargas-Rodriguez et al., 2018). The recent identification of two naturally orthogonal aaRS-tRNA pairs (PylRS-tRNA^Pyl^ or TrpRS-tRNA^Trp^) in the same organism suggests that additional co-existing orthogonal aaRS-tRNA pairs may be present (Mukai et al., 2017a; Castelle et al., 2018; Zhang et al., 2022).
